# Massive parallel sequencing in individuals with multiple primary tumours reveals the benefit of re-analysis

**DOI:** 10.1186/s13053-021-00203-z

**Published:** 2021-10-28

**Authors:** Karin Wallander, Håkan Thonberg, Daniel Nilsson, Emma Tham

**Affiliations:** 1 Department of Molecular Medicine and Surgery, Karolinska Institutet, Stockholm, Sweden; 2 Department of Clinical Genetics, Karolinska University Hospital, Stockholm, Sweden

**Keywords:** Hereditary cancer, Re-analysis, WGS, WES, Multiple primary, MEN1, MLH1

## Abstract

Multiple primary cancers, defined as three or more primary tumours, are rare, and there are few genetic studies concerning them. There is a need for increased knowledge on the heritability of multiple primary cancers and genotype-phenotype correlations. We have performed whole-genome/exome sequencing (WGS/WES) in ten individuals with three or more primary tumours, with no previous findings on standard clinical genetic investigations. In one individual with a clinical diagnosis of MEN1, a likely pathogenic cryptic splice site variant was detected in the *MEN1* gene. The variant (c.654C > A) is synonymous but we showed in a cDNA analysis that it affects splicing and leads to a frameshift, with the theoretical new amino acid sequence p.(Gly219Glufs*13). In one individual with metachronous colorectal cancers, ovarian cancer, endometrial cancer and chronic lymphocytic leukaemia, we found a likely pathogenic variant in the *MLH1* gene (c.27G > A), and two risk factor variants in the genes *CHEK2* and *HOXB13*. The *MLH1* variant is synonymous but has previously been shown to be associated to constitutional low-grade hypermethylation of the *MLH1* promoter, and segregates with disease in families with colorectal and endometrial cancer. No pathogenic single nucleotide or structural variants were detected in the remaining eight individuals in the study. The pathogenic variants found by WGS/WES were in genes already sequenced by Sanger sequencing and WES in the clinic, without any findings. We conclude that, in individuals with an unequivocal clinical diagnosis of a specific hereditary cancer syndrome, where standard clinical testing failed to detect a causative variant, re-analysis may lead to a diagnosis.

**Supplementary Information:**

The online version contains supplementary material available at 10.1186/s13053-021-00203-z.

## Introduction

Globally, cancer is the second most common killer and accounts for 16% of all deaths [1]. In Sweden, the most common types of cancer are prostate cancer, breast cancer, and non-melanoma skin cancer [2]. The risk of a second cancer in individuals who have already had one is higher than in the general population [3]. Multiple primary tumours are defined by the Surveillance Epidemiology and End Results (SEER) and the International Association of Cancer Registries (IACR) as two or more histologically distinct tumours, not caused by metastasis, recurrence, or local spread, and diagnosed in the same individual [4, 5]. 0.1–0.2% of all patients with cancer are estimated to have at least three different primary malignancies [6, 7], but reports on cohorts with three or more primary tumours are rare. In Sweden, 3% of all tumours registered between 2014 and 2018 were the third tumour discovered in that individual. It is important to note, however, that those are not necessarily primary tumours, since the numbers include recurrences and only synchronous tumours in the same organ had been excluded [8].

Multiple primary tumours can be caused by genetic predisposition, common exposure of carcinogenic agents, an underlying developmental abnormality, or chance. There are some already defined hereditary cancer syndromes that are associated with an increased risk of multiple primary tumours, such as Lynch syndrome and Li-Fraumeni syndrome [9]. However, we do not yet have full knowledge on the association between a specific pathogenic gene variant and all potential cancers that it can give rise to. An example of this is the *CHEK2* gene, wherein there are pathogenic variants that give rise to an increased risk for breast cancer but the extent of risks for other cancers is under debate [10, 11].

Knowledge on the genetic background of hereditary cancer is crucial for the treatment and follow-up of patients and their families. At clinical genetics departments, we offer genetic screening based on the combination of cancer types within the family. Usually, testing is done using a predefined cancer gene panel. For individuals with three or more primary tumours who do not fulfil clinical criteria, or in whom no causative variants are detected in clinical testing, there is no consensus concerning additional genetic testing. It has been suggested that whole-genome/exome sequencing (WGS/WES) should be considered for this patient cohort [12], but the clinical benefits of WGS/WES compared to standard clinical gene panels are arguable. Therefore, we performed WGS/WES in ten individuals with three or more primary tumours, to investigate if these techniques could identify novel germline genetic variants and thus provide additional clinical utility.

## Materials and methods

Participating families were recruited from the Department of Clinical Genetics at Karolinska University Hospital, Stockholm, Sweden, between 2013 and 2015. The inclusion criteria were three or more primary tumours with the first tumour before the age of 60 years (or four or more primary tumours at any age) in one individual in the family, with no obvious etiological cause. All participants had been clinically investigated for known cancer syndromes, either by targeted genetic analyses or by medical history.

Blood samples were collected in EDTA tubes and DNA was extracted according to standard procedures using Qiagen DNA extraction kit (QIAsymphony, DSP DNA Midi Kit, Hilden, Germany). WGS/WES was performed on the Illumina 2500 platform (San Diego, CA, USA) at the Department of Clinical Genetics and the Science for Life Laboratory, Solna Sweden, according to clinical procedures [13, 14] in 2015 and 2016. The first four included samples (from participant F, G, H and I) were analysed using WES, and for the other six samples (from participant A, B, C, D, E, and J) WGS was used. For WES, an average of 175 M reads were generated, for a 156x average coverage (98.1% of bases to 20x) in OMIM morbid genes [15, 16]. For WGS, an average of 861 M reads were generated, for a 37.0x coverage (99.0% of bases to 20x) in OMIM morbid genes. See Supplementary Table S1 for individual values. Read alignment, variant calling and variant annotation was performed with MIP v9.0.2 (https://github.com/Clinical-Genomics/MIP) and analysis and interpretation in Scout v4.29 (https://github.com/Clinical-Genomics/scout).

Three separate variant selection approaches were used, as can be seen in Supplementary Fig. S1. Fist, a custom filtering of all variants in the VCF file was applied. An in silico cancer gene list of 302 genes had been applied; a merge between a list of known somatic driver genes defined by Vogelstein et al. [17] and a local list of hereditary cancer associated genes, see Supplemental Table S2A. Only genetic variants with a maximal minor allele frequency (MMAF) lower than 0.1% in ExAC [18] and with an allele fraction (alternate/(reference + alternate) allele ratio) above 30% were considered. All variants known to be pathogenic/likely pathogenic for a well-defined hereditary cancer syndrome according to ClinVar [13] [19], were included and variants reported to be benign by more than one source were excluded. In addition, exonic or splice variants were included, and synonymous variants were only included if they had a predicted effect on splicing by SpliceSiteFinder-like, MaxEntScan, GeneSplicer, and NNSPLICE [20]. The predictions on protein effect were extracted from Align GVGD [21] [22], SIFT [23], PolyPhen2 [24], and Mutation Taster [25]. The Alamut software (Alamut Visual, Interactive Biosoftware, Rouen, France) was used to access and visualise the predictions. It was also noted if the variant had been reported as a somatic cancer mutation in the database cBioPortal [26]. The ACMG (American College of Medical Genetics and Genomics) classification system was followed for pathogenicity annotation for all filtered variants [27]. We also checked if there were specific adjustments to the ACMG criteria stipulated by the Clinical Genome Resource ClinGen for the genes investigated [28]. Heterozygous variants judged to be pathogenic according to the ACMG criteria, but not considered disease-causing in the participant, were classified as pathogenic incidental findings (for autosomal recessive conditions) or risk factors.

Secondly, the variants were ranked using the software Genmod (https://github.com/moonso/genmod) and visualized in Scout [29]. An in silico gene list related to the human phenotype ontology term HP:0002664 Neoplasm (accessed on 11-25-2019) and a curated gene list from the Department of Clinical Genetics (Supplementary Table S2B and S2C) were used. Scout allows variant triage by display of ranked variant lists with relevant annotation information, such as population frequency, local variant frequency, previous clinical classification in e.g. ClinVar, conceptual functional annotation by VEP and common variant effect prediction scores including CADD, SIFT and PolyPhen [30] [23] [24]. All variants were examined in two analyses, one predicting a dominant disease and the other searching for variants associated to a recessive disease (homozygous and compound heterozygous variants). Genetic sequence variants were excluded if they were classified as benign/likely benign by ClinVar by multiple sources [19], if they occurred in a non-coding, non-splice-site region, if they were reported to have occurred more than three times in another cohort (among 4755 individuals tested with WGS/WES locally) or had a MMAF > 0.1% globally [18]. Variants in a gene known to cause a specific syndrome were considered less relevant if the individual harbouring that variant had no clinical features of that syndrome. The selected variants were ranked according to the same criteria as the variants from the initial cancer gene lists, and classified using the ACMG criteria.

Thirdly, we created a shortlist of genes which were specifically interesting in each participant according to their cancer diagnoses and their family history (see Supplementary Table S3). We checked all variants including non-coding/synonymous variants in those genes. Sequence variants were excluded if they were classified as benign/likely benign by ClinVar by multiple sources [19], or had a MMAF > 0.1% globally [18]. Non-coding variants were checked for occurrence in the Blueprint genetic list of non-coding variants included in their genetic hereditary cancer screening test [31].

Finally, we used Scout to find structural variants (SVs) in the data from the six individuals who had had a WGS analysis. Scout uses four SV callers: Delly, Manta, CNVator and TIDDIT [32–35] and filters against a reference database with 1000 Swedes [36] and our local database of 4755 individuals. The in silico cancer gene list from the Department of Clinical Genetics, Karolinska, (Supplementary Table S2C) was used and all SVs were examined. Variants were excluded if they occurred in more than five persons in the local database, or were likely artefacts (either occurring in repetitive genome areas and/or large copy number variants called by primarily discordant read pair focused callers (Manta, Delly) [33] [32] and not supported by read coverage (as e.g. CNVnator) [34]). As with single nucleotide variants, variants that were not predicted to modify expressed gene products (e.g. intronic duplications or inversions with intergenic breakpoints) were dismissed from further triage.

For expression analysis of the *MEN1* gene, blood was collected from participant E in a PAXgene® Blood RNA Tube (PreAnalytiX GmbH, Switzerland) and RNA was isolated with PAXgene Blood RNA Kit accordingly to the manufacture’s instructions (PreAnalytiX GmbH, Switzerland). Subsequently, cDNA was prepared with reverse transcriptase SuperScript® VILO™ MasterMix (Thermo Fisher Scientific, MA USA), and sequenced with Sanger sequencing using specific primers; forward 5′-CGTGAGCTGGTGAAGAAGGT-3′, and reverse, 5′-GTCCCAGGTCATAGAGCAGC-3′. PCR was performed with AmpliTaq Gold (ThermoScientific, US) with the following cycle-program: 96 °C 10 min, (96 °C 30 s, 62 °C 30 s, 72 °C 40 s) × 35, 72 °C 8 min.

## Results

Ten individuals with three or more primary tumours were included in the study, eight women and two men. The participants’ phenotypes are listed in Table 1.

**Table 1:** Medical history of the included participants

Participant	Sex	Age at time of study	First cancer location (age)	Second cancer location (age)	Third cancer location (age)	Fourth cancer location (age)	Cancer in first degree relatives?	Chemotherapy? /Radiation therapy?	Anti-oestrogen treatment?	Smoking regularly > 5 years	Genetic tests performed before inclusion in the study (normal results unless stated otherwise) (c)
A	W	63	Adenocarcinoma colon (30, 55)	Adenocarcinoma ovarium (47)	Adenocarcinoma endometrium (47)	CLL (55)	Yes	NA/NA	NA	NA	*MLH1, MSH2, MSH6, EPCAM, PMS2.* Tumour tissue BRAF neg. IHC MMR: loss of MLH1 and PMS2.
B	W	46	Teratoma ovaries (30)	Rectum (33)	Breast (40)	NA	No	Yes/Yes	Yes	NA	*MLH1, MSH2, MSH6, EPCAM, PMS2, BRCA1, BRCA2*
C	W	75 (a)	Breast (60)	Colon (62)	Lung (75)	NA	Yes	Yes/Yes	No	Yes	*BRCA1, BRCA2, TP53.* IHC MMR normal.
D	W	66 (a)	Malignant melanoma (59)	Breast (63), tubular	Endometrium (64)	Pancreas (65)	Yes	Yes/Yes	Yes	No	*CDKN2A, BRCA1, BRCA2, TP53, PALB2, CHEK2, ATM, CDK4*
E	W	38	Adrenal adenoma (27)	Parathyroid hyperplasia (27)	Pituitary adenoma - prolactin and GH producing (28)	Multiple neuroendocrine tumours in pancreas (30)	Yes	No/No	No	No	Endocrine tumour gene panel (d)
F	W	75	Breast (59), multifocal ductal	Melanoma in situ (62, 70)	Endometrium (64)	Basalioma (76) (b)	Yes	No/No	No	Yes	*PTEN.* IHC MMR normal.
G	M	74	Adenocarcinoma lung, 3 primaries (54, 69, 70)	Prostate (56), Gleason 7	Rectum (57)	Carcinoid caecum (57)	No	No/Yes	Yes	No	Karyotype
H	W	76	Cervix (41)	Breast (59, 69), lobular	CLL (63, 68)	Adrenal cortical carcinoma (67)	Yes	No/Yes	Yes	No	*BRCA1, BRCA2, CHEK2, TP53, PTEN, STK11, CDH1.* CGH-array
I	M	76	Prostate (57), Gleason 7	GIST oesophagus (68)	Colon (68)	NA	Yes	Yes/Yes	No	No	
J	W	74	Endometrium (41)	Synovial sarcoma (54)	Breast (61), ductal	NA	Yes	No/Yes	No	Yes	*BRCA1, BRCA2, CHEK2, TP53, PTEN, STK11, CDH1*

(a) deceased at the time of study

(b) diagnosed after inclusion

(c) genetic testing by either Sanger sequencing, Ion Torrent massive parallel sequencing or WES, with deletion/duplication testing using multiple ligation-dependent probe amplification or targeted CGH-array

(d) WES, in silico gene panel including: *AIP, AKT1, AP2S1, ARMC5, CACNA1A, CACNA1H, CASR, CDC73, CDH23, CDKN1A, CDKN1B, CDKN2B, CDKN2C, CLCN2, CYP11B1, CYP11B2, FH, GNA11, GNAS, GPR101, KCNJ5, KIT, KLLN, MAX, MEN1, MET, MLH1, MSH2, MSH6, NF1, PDE11A, PDE8B, PDGFRA, PIK3CA, PMS2, PRKAR1A, PTEN, RET, SDHA, SDHAF2, SDHB, SDHC, SDHD, TMEM127, TP53, TSC1, TSC2, USP8, VHL*

*W* Woman, *M* Man, *CLL* Chronic lymphocytic leukaemia, *IHC MMR* Immunohistochemistry of mismatch-repair genes, *GIST* Gastrointestinal stroma tumour, *NA* No information available

Two participants, A and E, fulfilled the clinical criteria for specific hereditary cancer syndromes. Participant A had metachronous cancers in the colon (with defect mismatch repair), ovaries, and uterus, with her first cancer at the age of 30. Her father had colorectal cancer at the age of 76. This is highly suggestive of Lynch syndrome or Lynch-like syndrome. Participant E fulfilled the clinical criteria for MEN1 syndrome (multiple endocrine neoplasia type 1) [37], with parathyroid hyperplasia (at 27 years of age), prolactin and GH producing pituitary adenoma, and multiple neuroendocrine tumours in the pancreas, all of them less than one cm. She also had a malignant melanoma of 1.4 mm at the age of 33. Her mother was operated for primary hyperparathyroidism at 47 years of age. She also had a prolactin-producing microadenoma of the pituitary and multiple lipomas.

Four participants had possible hereditary cancer syndromes, based on age of cancer onset or occurrence of multiple tumours known to be associated to the same syndrome. Participant B had a suspected familial colorectal cancer syndrome. Participant C and H both had suspected familial breast and ovarian cancer syndromes. Participant C had a mother with ovarian cancer at the age of 57, a sister with breast cancer at the age of 62, and a father with prostate cancer at the age of 61, and participant H had a brother with testicular cancer at the age of 36. The clinical criteria for familial malignant melanoma [38, 39] were met in the family of participant D, who had malignant melanoma at 59 years of age, and a brother with malignant melanoma at 40 years of age.

The remaining four participants did not fulfil any hereditary cancer syndrome criteria. In the family of participant I, both the brother and the father had prostate cancer at 65 and 80 years of age, respectively, suggestive of familial prostate cancer. Participant J had early onset uterine cancer, but unfortunately, no tissue biopsy was available and no microsatellite instability or immunohistochemistry analysis for mismatch-repair proteins could be performed. Her family history was not suggestive of Lynch syndrome.

In the ten participants, a total of 32 potential cancer syndrome candidate single nucleotide variants were identified from WGS/WES, see Table 2. All of the variants occurred in a heterozygous state and a majority were missense. No structural variants relevant for cancer were detected.

**Table 2:** All selected variants

Participant	Gene symbol	Refseq	Nucleotide substitution	Predicted protein alteration	VAF (a)	cBioportal (b)	gnomAD	Variant interpretation
A	*CHEK2*	NM_001005735.1	1229delC (1100delC)	Thr410Metfs*15	0.4	Ab	0.008717	Risk factor
A	*SDHA*	NM_004168.2	1724C > T	Ala575Val	0.4	Bc	0.0001386	VUS
A	*ABCB11*	NM_003742	1724G > A	Arg575Gln	0.4	Bb	0.0003315	VUS
A	*HOXB13*	NM_006361	251G > A	Gly84Glu	0.5	Ac	0.007618	Risk factor
**A**	***MLH1***	**NM_000249**	**27G > A**	**Arg9=**	**0.5**	**Ba**	**0**	**Likely pathogenic**
B	*CHEK2*	NM_001005735.1	670C > T	Arg224Cys	0.7	Ab	0.0005227	VUS
B	*KLLN*	NM_001126049.1	422G > A	Thr141Met	0.6	C	0	VUS
B	*REST*	NM_005612	968 T > G	Met323Arg	0.5	Ac	0	VUS
B	*TRIM28*	NM_005762	2381C > T	Thr794Met	0.5	Ab	0.0001986	VUS
B	*LZTR1* (c)	NM_006767	1866_1867delTC	Pro623ThrfsTer45	0.5	Bb	0	VUS
C	*BAP1*	NM_004656.3	944A > C	Glu315Ala	0.4	Bb	0.0003263	VUS
C	*FBXW7*	NM_001013415	248G > A	Arg83Lys	0.5	Bc	0.0005178	VUS
C	*RMRP* (c)	NR_003051	71A > G	NA	0.4	C	0.008687	Pathogenic incidental finding
C	*SMO*	NM_005631.4	517C > T	Arg173Cys	0.5	Bb	0.0007454	VUS
E	*ARID1B*	NM_020732.3	4727C > T	Pro1576Leu	0.4	Ac	0.00003098	VUS
E	*MLH1*	NM_000249.3	41C > T	Thr14Ile	0.7	Bc	0.000008792	VUS
E	*SSX1*	NM_005635	293dupA	Met99AspfsTer24	0.6	Bc	0.0002149	VUS
E	*FAT4*	NM_024582	7751C > A	Ser2584Tyr	0.5	Bc	0.00005789	VUS
E	*TOP2A*	NM_001067	154G > A	Gly52Ser	0.4	Bc	0	VUS
**E**	***MEN1***	**NM_000244**	**654C > A**	**Ala213=**	**0.6**	**Bc**	**0**	**Likely pathogenic**
F	*TYR* (c)	NM_000372.4	1147G > A	Asp383Asn	0.6	Ab	0.0001784	Pathogenic incidental finding
G	*ERCC3* (c)	NM_000122.1	1204G > A	Gly402Ser	0.5	Bc	0.00004006	VUS
G	*CTNNB1*	NM_001098210.1	319A > G	Met107Val	0.5	Ac	0	VUS
G	*TCF3*	NM_003200	689C > G	Pro230Arg	0.5	Bc	0.00001430	VUS
H	*RNF6*	NM_005977	1780C > A	Leu594Ile	0.4	Bc	0	VUS
H	*RNF6*	NM_005977	895G > C	Glu299Gln	0.3	Bc	0.000008792	VUS
H	*ARHGAP26*	NM_015071	619C > G	Leu207Val	0.5	Bc	0.00001760	VUS
H	*XRCC3*	NM_005432	172C > T	Arg58Trp	0.5	Ac	0.00006208	VUS
H	*TSC2*	NM_000548	1244C > T	Ala415Val	0.5	Ac	0.0001866	VUS
J	*TCF3*	NM_003200	c.1213C > T	p.Arg405Cys	0.5	Ac	0.00008796	VUS
J	*PNP* (c)	NM_000270	c.701G > C	p.Arg234Pro	0.6	C	0.0001471	Pathogenic incidental finding
J	*ARHGAP26*	NM_015071	c.1559A > G	p.Gln520Arg	0.5	C	0.00006175	VUS

*VUS* Variant of unknown significance

(a) VAF; variant allele fraction (alternate/(reference + alternate))

(b) cBioPortal [26]:

A: Same variant

B: Other similar variant in proximity

C: No similar variant in proximity

a: Same tumour types

b: Overlapping tumour types

c: Other tumour types

(c) Gene associated to potentially cancer-associated syndrome described in OMIM (Online Inheritance in Man) [40] with a recessive inheritance pattern, including those with both recessive and dominant inheritance patterns

**Fig. 1:**
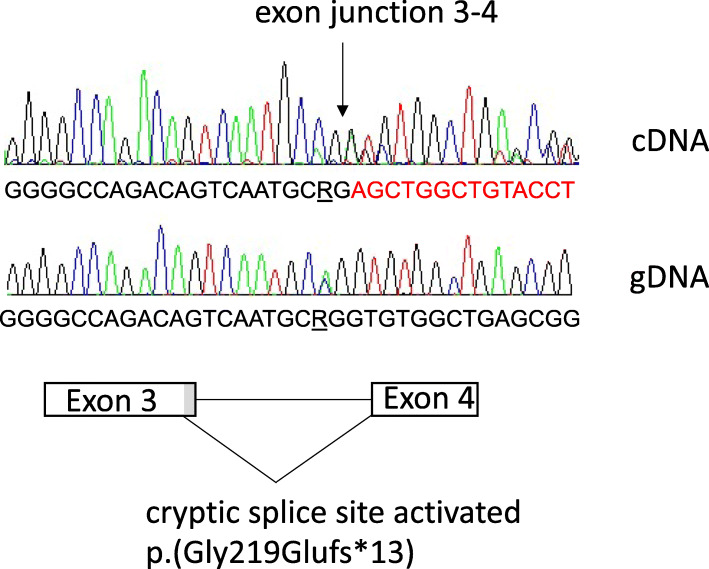
Effect of the *MEN1* variant on splicing

A clinically highly relevant synonymous variant was found in the *MEN1* gene in participant E, with a clinical diagnosis of MEN1. Her DNA had been sequenced twice via the Department of Clinical Genetics at Karolinska University Hospital (using specific Sanger sequencing and MLPA of the *MEN1* gene in 2009, and whole-exome sequencing and an in silico gene list for endocrine tumours in 2015), with no pathogenic variant found (Table 1). The synonymous c.654C > A *MEN1* variant was detected in the clinical testing, but it was classified as likely benign and not mentioned in the report. The variant had been reported as a likely benign variant in ClinVar (variation ID 748511, accessed 07-03-2021), by a single submitter and without further details [19]. However, in the present analysis, the variant was predicted by the Alamut software to potentially strengthen a cryptic splice site (SpliceSiteFinder-like from 71.5 to 80.4, MaxEntScan, 5 to 6.3, GeneSplicer, 4.3 to 7.6, and NNSPLICE 0 to 0.8) [20], leading to a deletion of 14 nucleotides in exon 3 and a frameshift in the RNA sequence (Fig. 1). The variant is not present in a reference database (gnomAD v2.1.1) [41]. The variant was therefore investigated further by cDNA sequencing. This revealed that the individual had a frameshift, as predicted, and the conceptual new amino acid sequence p.(Gly219Glufs*13). Based on the information from the functional analysis, the variant fulfilled the specific ACMG criteria PS3, PM2 and PP4 and it was classified as likely pathogenic; with a spliceogenic loss-of-function effect [27]. The family members had been tested for MEN1 biochemically before this study, and the mother was diagnosed with a primary hyperparathyroidism and underwent surgery at the age of 47. The *MEN1* variant was inherited from the affected mother and absent from the healthy father, and the 38-year-old healthy sister.

The sequence reads from cDNA and gDNA from participant E, followed by a depiction of the activation of a cryptic splice site 14 nucleotides upstream of the normal splice site at the 3′ end of exon 3. The novel splice site leads to a loss of 14 nucleotides and a frameshift on the mRNA level.

Using the approach of targeting genes associated to the participants’ specific cancer diagnoses yielded one more likely pathogenic variant: the *MLH1* variant in participant A. The variant had been detected by Sanger sequencing performed at the Department of Clinical Genetics, Karolinska University Hospital, in 2012, but as it was synonymous and another nucleotide change at the same position was common in gnomAD, it was considered likely benign. The variant was not predicted to affect splicing by the Alamut software. When the variant was found in our study, though, it had been reported in ClinVar as likely pathogenic by two sources (variation ID 186982, accessed 07-03-2021) [19] [41]. The variant fulfilled the specific ACMG criteria PS4, PM2 and PP4 [27]. Therefore, it was considered likely pathogenic. Immunohistochemistry of participants A’s colon cancer biopsy demonstrated loss of protein expression of MLH1 and PMS2, indicative of *MLH1* loss of function, and no *BRAF* V600 mutation was found in tumour DNA.

Two known pathogenic moderate cancer risk variants were also found in participant A; in the genes *CHEK2* and *HOXB13* (Table 2). In the rest of the participants, no pathogenic variant was found.

Three individuals were heterozygous carriers of variants that are known to cause autosomal recessive conditions if found in a homozygous or compound heterozygous state. These variants, in the genes *RMRP*, *TYR*, and *PNP,* were classified as incidental findings.

Participant G carried a rare *CTNNB1* variant, p.(Met107Val). In order to determine if this variant affected the protein expression of catenin B, immunohistochemistry analysis was performed on tumour tissue from participant G at the Department of Clinical Pathology, Karolinska University Hospital, Sweden. A normal pattern of catenin B staining in the cell membranes was found in tumour tissue from the prostate, rectum and initial lung tumour, with a weaker signal in the neuroendocrine tumour. The two later lung tumours demonstrated both membranous and cytoplasmic staining with some nuclear staining in the adenocarcinoma of the right lung.

No variants remained after filtering for participants D and I.

## Discussion

Most publications concerning predisposition to multiple primary tumours include predominantly individuals with two primary malignancies and reports on cohorts with three or more are rare. Multiple primary tumours may be caused by a monogenic predisposition, but may also be due to multifactorial risk factors. There is no clinical consensus on which genetic investigation is appropriate for patients with three or more primary tumours. It is clear that more knowledge is needed before clinical criteria for testing in this patient cohort can be stipulated. We chose to only include individuals with at least three different primary tumours in this study in order to select a rare group (incidence 0.1–0.2% [6] [7]) and to minimize the risk of only including secondary therapy-related cancers.

The WGS analysis led to the detection of a coding variant in the *MEN1* gene in participant E. Multiple endocrine neoplasia syndrome 1 (MIM #131100) is characterised by a predisposition to many different endocrine neoplasms, in particular of the parathyroid, pancreas, and anterior pituitary gland. It is inherited in an autosomal dominant fashion and caused by pathogenic, mostly truncating, variants in the tumour suppressor gene *MEN1* [37, 42]. 46 of 356 known pathogenic/likely pathogenic variants in *MEN1* are splice site variants [19, 43]. Participant E had an established clinical diagnosis of MEN1, with three major manifestations as well as an adrenal gland adenoma. Although the variant had been reported as likely benign once in ClinVar (variation ID 748511, accessed 07-03-2021), it was not present in a reference population and was predicted to affect splicing. Therefore, we further investigated the functional effects of the variant. cDNA sequencing confirmed that the variant causes a frameshift on the RNA level and can thus be classified as class 4/likely pathogenic by ACMG criteria. The pathogenicity is further supported by the fact that the mother of participant E, who also had MEN1 clinically, is a carrier of the variant and the healthy sister and father are not. Although we detected this variant by WGS, it was not the WGS per se that enabled the finding, but rather a thorough investigation of the coding sequence of the *MEN1* gene. There are suggestions on adjustments of the ACMG criteria for *MEN1* variant annotations, with more emphasis on the clinical picture and family history [44]. This further underlines the need for evaluating the variant in light of the participant’s syndrome, and not excluding variants with a benign reputation. Indeed, in 10–30% of patients with a clinical diagnosis of MEN1 syndrome, no pathogenic *MEN1* variant is found, and it has been suggested that re-analysis of these patients might be beneficial [45]. In a recent study by Backman et al., three out of 14 patients with MEN1 syndrome, but no pathogenic *MEN1* variant found on clinical genetic testing, actually did carry a pathogenic variant when re-analysed [46]. The other likely pathogenic variant found in this study, the c.27G > A *MLH1* variant in participant A, was found by the approach of including all rare synonymous variants in candidate genes, regardless of their effect on splicing. The variant found in this study has been found in a study performed by Ward et al., in two individuals: one with early onset (22 years), the other with multiple colorectal cancers (50, 52 and 54 years), both with MLH1 loss on immunohistochemistry analysis and low-level constitutional hypermethylation of the *MLH1* promoter. Two family members with colorectal cancer at older age (49 and 61 years) did not carry the variant, but the microsatellite status of their tumours was unknown [47]. The same variant has also been described by Leclerc et al., in a family fulfilling the Amsterdam I criteria. Low-level constitutional *MLH1* hypermethylation and the c.27G > A variant segregated with disease within the family. It was also found on a different haplotype in an individual with colorectal and endometrial cancer from another family [48]. In the ClinVar entry for the present variant, Ambry Genetics state that low-level *MLH1* promoter methylation and the c.27G > A variant segregate with Lynch syndrome in their internal data material and that their RNA studies have shown the variant does not cause abnormal splicing (variation ID 186982, accessed 07-03-2021) [19]. The variant was classified as likely pathogenic in our study. It is again clear that re-evaluation and not the WGS per se was the most important aspect of this discovery. Of note, participant A had in total five different primary tumours: two colonic, one endometrial, one ovarian, and a chronic lymphocytic leukaemia.

Thus, if a patient fulfils clinical criteria of a specific cancer syndrome it is worthwhile to re-examine the clinical WGS/WES data for rare synonymous/splice site variants. The benefits of re-analysis of genomic data have been previously shown, for instance in cohorts of children with developmental disabilities or congenital syndromes [49, 50]. Patients with a high suspicion of a specific hereditary cancer syndrome will likely also benefit from periodic re-analysis. Stafford et al. emphasize the importance of re-analysis of genes involved in known tumorigenesis molecular pathways in ovarian cancer patients [51] but further reports on WGS/WES re-analysis in cancer cohorts are so far rare. There is a need for more knowledge on the advantages and disadvantages of this approach in the clinical setting. Similarly, there could potentially be variants in this study that end up being classified as pathogenic in the future.

Also, in participant A, two variants classified as risk factors were discovered in *CHEK2* and *HOXB13*. *CHEK2* is considered to harbour moderate-penetrance cancer-susceptibility genetic variants, and the most well-studied is the variant found in our study. It is commonly called 1100delC, p.(Thr367fs), but with the most recent nomenclature named c.1229delC, p.(Thr410fs). The truncating c.1229delC variant is known to be a moderate-risk variant for hereditary breast cancer, with the relative risk estimated to be 3.0, and a cumulative risk for breast cancer by 80 years of age of 32% [52]. In clinical practice in Sweden today, female carriers of truncating *CHEK2* variants, who also have a first degree relative with breast cancer, are offered annual mammograms at ages 40 to 60 [53] [54]. Guidelines differ internationally though, and the NCCN (National Comprehensive Cancer Network) recommend all female *CHEK2* truncating variant carriers, regardless of family history, to undergo annual screening, in some cases with breast MRI and contrast, starting at or before 40 years of age [55]. In the United Kingdom, annual mammography at ages 40 to 50 is recommended for all carriers of truncating variants in the *CHEK2* gene, regardless of family history [56]. Truncating *CHEK2* variants have also been shown to confer a slightly increased risk for gastric cancer, kidney cancer, sarcoma, prostate cancer [10], and colorectal cancer [11], with the relative risk for the latter estimated to be 1.88 [52]. These risks are too low to be used to recommend surveillance in the clinic. Participant A’s cancers are all likely caused by the Lynch syndrome variant; the *CHEK2* variant is less likely to impact on her phenotype. Since participant A did not have any known relative with breast cancer, she and her close relatives were not recommended breast cancer surveillance and no predictive *CHEK2* testing could be performed in her family.

Missense variants in the *CHEK2* gene may confer a small increase in the risk of cancer, but robust data is only available for the missense variant c.599 T > C, which leads to an estimated relative risk of breast cancer of 1.58 and of colorectal cancer of 1.56 [52]. Individual assessment is recommended for non-truncating *CHEK2* variants [55]. The *CHEK2* variant c.670C > T (historically, and more commonly known as NM_007194.4:c.541C > T, p.(Arg181Cys)) found in participant B, has been classified as of unknown significance by multiple submissions to ClinVar (variation ID 5597, 24-03-2021) [19]. It is situated in the forkhead-associated (FHA) domain [57], just as the c.599 T > C variant. Disturbances of this region can obstruct the binding of CHEK2 to BRCA1 [58]. The c.670C > T variant has been reported in individuals with breast cancer [57, 59] and colorectal cancer [60]. Participant B had rectal and breast cancer at a young age and one of her relatives had had gastric cancer. Although this variant has likely contributed to participant B’s cancer risk, the risk contribution is probably small and would not lead to any special surveillance. Thus, the variant is considered of unknown significance and predictive testing cannot be offered in the family.

The variant in the *HOXB13* gene, c. 251G > A, found in participant A, is listed in ClinVar as pathogenic/likely pathogenic/risk factor (variation ID 128031, 24-03-2021) [19]. It is known to increase the risk for prostate cancer, with carriers having an estimated 4.5 times higher risk of prostate cancer than non-carriers [61, 62]. It has also been suggested to increase the risk for colorectal cancer, with a significant association between the *HOXB13* variant and colorectal cancer (OR 2.8) [63]. However, it does not increase the risk for breast cancer [64], and its potential role as a germline risk factor for other cancers is not proven. In Sweden, the Regional Cancer Committee advises against routinely testing for this *HOXB13* variant in families, since individuals not carrying the variant cannot be dismissed from clinical screening for prostate cancer, and the consequences of testing family members are not yet known [65]. The NCCN does not specify management of *HOXB13* pathogenic variant carriers but acknowledges it as a variant associated to an increased risk for prostate cancer [66]. The Philadelphia Prostate Cancer Consensus has proposed that all male *HOXB13* pathogenic variant carriers are offered surveillance with PSA tests from not later than 40 years of age [67]. Participant A is a woman, and no clinical action will be taken upon the finding. Her father had prostate cancer at an age of 78 and it is reasonable to believe the variant could have contributed to this.

In participant B, a missense variant in the gene *KLLN* was also present. Hypermethylation of the *KLLN* promoter is potentially causative for Cowden syndrome, which includes an increased risk for breast and colorectal cancer [68]. Participant B had indeed breast and rectal cancer, in addition to teratoma. The missense variant found in this study has not been reported before and is of unknown significance as of today.

In addition to the above, three individuals were found to be healthy carriers of pathogenic heterozygous variants that cause autosomal recessive disease (Table 2). None of these are clinically actionable in the heterozygous state and would therefore not be reported in Sweden.

Participant H had ongoing chronic lymphocytic leukaemia at the time of the study, and DNA isolated from the blood sample could therefore contain leukemic cells (around the time of DNA isolation from peripheral blood the leukocyte particle concentration was 20 × 10^9^/L). Several of the detected variants were below our cut-off variant allele fraction of 30%, and were therefore excluded. Among the variants in Table 2, two variants in the *RNF6* gene had variant allele fractions of 40 and 32%, respectively, and are likely also somatic. The *RNF6* gene has no known connection to inherited cancer [40].

No other likely pathogenic variants were detected. We have not analysed more common genetic risk factors. Also, we cannot rule out environmental factors contributing to the participant’s cancers. Chemotherapy, hormone therapy, smoking and exposure to UV light are all known cancerogenic agents that may explain part of the clinical phenotype, but are unlikely to be responsible for the full clinical picture of the participants in this study. For example, participant D had received Tamoxifen, an anti-oestrogen treatment for her breast cancer. Tamoxifen treatment increases the risk of endometrial cancer, especially after longer use (> 2 years) [69]. Patient D developed endometrial cancer within 1 year after diagnosis of her breast cancer, and it is difficult to know if it was induced by the Tamoxifen or not. Of note, she had two additional primary tumours (malignant melanoma at 59 years and pancreatic carcinoma at 65 years) which cannot be attributed to Tamoxifen.

There are few studies analysing individuals with three multiple primaries. Whitworth et al. recently performed whole-genome sequencing in a cohort of 460 individuals with two or three primary tumours [12], of whom 182 had at least three multiple primaries (personal communication). Of those, 31 (17%) harboured pathogenic or likely pathogenic variants. 30 of the 31 individuals with pathogenic or likely pathogenic variants had their first cancer diagnosed before the age of 60. No information on family history was provided. Six individuals with breast cancer harboured risk factor variants in the *ATM* or *CHEK2* genes, and if they had close relatives with breast cancer, they would have been offered clinical genetic testing according to guidelines. Of interest, two of the detected variants were in genes rarely analysed in cancer syndromes (*PTEN* and *NTHL1*), which may easily be missed using smaller clinical gene panels. This suggests that in cases with three or more primary tumours, a broad cancer gene panel might be recommended. Once genetic testing is expanded beyond clinical criteria, there is always a risk of additional findings. Indeed, at least three of the findings by Whitworth et al. in patients with three or more primary tumours (in the genes *BRCA2* and *MSH2*) may be classified as secondary, as they were found in individuals who had not had breast/ovarian or colorectal/endometrial cancer. Although European guidelines recommend using targeted panels to avoid incidental findings [70], several countries, including the UK [71] and France [72], have started to search for pathogenic variants in defined cancer genes after informed consent in all patients undergoing WES/WGS. In Sweden, informed consent for incidental findings is collected as part of research projects such as this one. Incidental findings in the clinical laboratory are reported after ethical review, but we do not actively search for causative variants in genes that are not known causes of the patient’s clinical syndrome.

Our study is not large enough to give any significant results but it supports the data from Whitworth et al. [12]: the genetic cause in patients who fulfil clinical criteria for specific cancer syndromes may be missed by conventional testing. This is either because the patient has a genetic variant which is difficult to identify (such as the *MEN1* or *MLH1* variant in our study), or because the syndrome is not recognised by the clinicians and therefore not tested for. One example is the *NTHL1* variant in an individual with meningioma and colorectal cancer in the Whitworth study [12]. Another is a patient with a pathogenic variant in *PTEN* discovered by WES in a young-onset colorectal cancer cohort. Cowden syndrome could be confirmed clinically when the medical history was re-examined [73]. A simple re-analysis of clinical WGS/WES data would have been sufficient to diagnose 2/10 individuals (20%) with pathogenic variants in our study. Potentially, though, we could have missed variants in rare genes that are not included in the clinical panel and that cause syndromes clinicians do not recognise.

In 8/10 (80%) of the individuals in this study, no monogenic cause of cancer was found. Four participants did not fulfil clinical criteria for any specific cancer syndrome. Multiple tumours in patients who do not fulfil testing criteria for hereditary cancer syndromes are most likely multifactorial. There might be genetic predisposition of lower penetrance in the individuals in the cohort, since we found possibly disease-related variants in cancer driver genes. WGS analysis for research purposes will probably detect interesting risk factors, including risk variants in genes such at *CHEK2* and *ATM*, but the clinical use of WGS in these cases is still debatable. WGS/WES also has technical limitations when it comes to analysis of genes with pseudogenes and nucleotide repeat regions. Although WGS can in theory detect all disease-causing variants, some types of pathogenic variants will be missed. This may be due to lack of information on normal variation of for instance non-coding variants, or due to difficulties in detecting complex variants, such as Alu repeat insertions, using conventional bioinformatics.

In summary, we have performed WGS/WES in ten individuals with three or more primary tumours, with previously normal results from clinical genetic testing. One of the participants, with clinical Lynch-like syndrome, harboured a synonymous variant in the *MLH1* gene, causing constitutional low level hypermethylation, which was considered likely pathogenic. Another participant had a clinical diagnosis of MEN1, and this study could identify a likely pathogenic cryptic splice variant in the *MEN1* gene*.* No clinically actionable variants were detected in the other eight participants. As rare hereditary cancer syndromes are difficult to identify in routine clinical practice, WGS/WES may provide additional benefit by including a wider gene list and also by potentially being able to detect rare structural variants that may cause cancer. We therefore recommend that individuals with three primary tumours, and with their first cancer diagnosed before 60 years of age, are referred to a clinical genetics department for an evaluation. From our findings, we conclude that if a patient fulfils clinical criteria for a specific cancer syndrome, a re-evaluation of the genetic variants in genes with known association to that syndrome might be warranted after a few years.

## Supplementary Information

**Additional file 1: Supplementary Table S1.** Sequencing coverage and reads information for each sample.

**Additional file 2: Supplementary Table S2A.** Gene lists; Hereditary cancer and Cancer driver. **Supplementary Table S2B.** Gene list; Scout HPO Neoplasia. **Supplementary Table S2C.** Gene list; Scout curated Clinical Genetics.

**Additional file 3: Supplementary Table S3.** Genes targeted in extra analysis for each participant.

**Additional file 4: Supplementary Fig. S1.** Schematics of the workflow for variant filtration.

## Data Availability

The relevant datasets generated during and analysed during the current study are available from the corresponding author on reasonable request. All computational analysis can be reproduced with freely available, open source code tools: MIP (https://github.com/Clinical-Genomics/MIP) and Scout (https://github.com/Clinical-Genomics/scout).
