# Correction to: Massive parallel sequencing in individuals with multiple primary tumours reveals the benefit of re-analysis

**DOI:** 10.1186/s13053-021-00204-y

**Published:** 2021-11-16

**Authors:** Karin Wallander, Håkan Thonberg, Daniel Nilsson, Emma Tham

**Affiliations:** 1 Department of Molecular Medicine and Surgery, Karolinska Institutet, Stockholm, Sweden; 2 Department of Clinical Genetics, Karolinska University Hospital, Stockholm, Sweden

**Correction to: Hered Cancer Clin Pract 19, 46 (2021)**

**https://doi.org/10.1186/s13053-021-00203-z**

Following publication of the original article [1], it was identified that in the author panel, the given names and family names were erroneously transposed. The correct names are as follows:

Karin (given name) Wallander (family name)

Håkan (given name) Thonberg (family name)

Daniel (given name) Nilsson (family name)

Emma (given name) Tham (family name)

Further to this, during production, corrections to the order of the references were mistakenly not implemented. The publishers apologize for this error and the references have been corrected.

In the Results section of the article, second paragraph, 14 cm should be changed to 14 mm. The sentence should read as follows: “She also had a malignant melanoma of 1.4 mm at the age of 33.

The original article [1] has been corrected.
