# Supplementary material for: Massive parallel sequencing in individuals with multiple primary tumours reveals the benefit of re-analysis
Source: Hered Cancer Clin Pract. 2021 Oct 28;19:46. doi: 10.1186/s13053-021-00203-z (PMC8555269; doi:10.1186/s13053-021-00203-z)
Supplement: Supplementary file 4 — Additional file 4: Supplementary Fig. S1. Schematics of the workflow for variant filtration. [file 13053_2021_203_MOESM4_ESM.pdf]

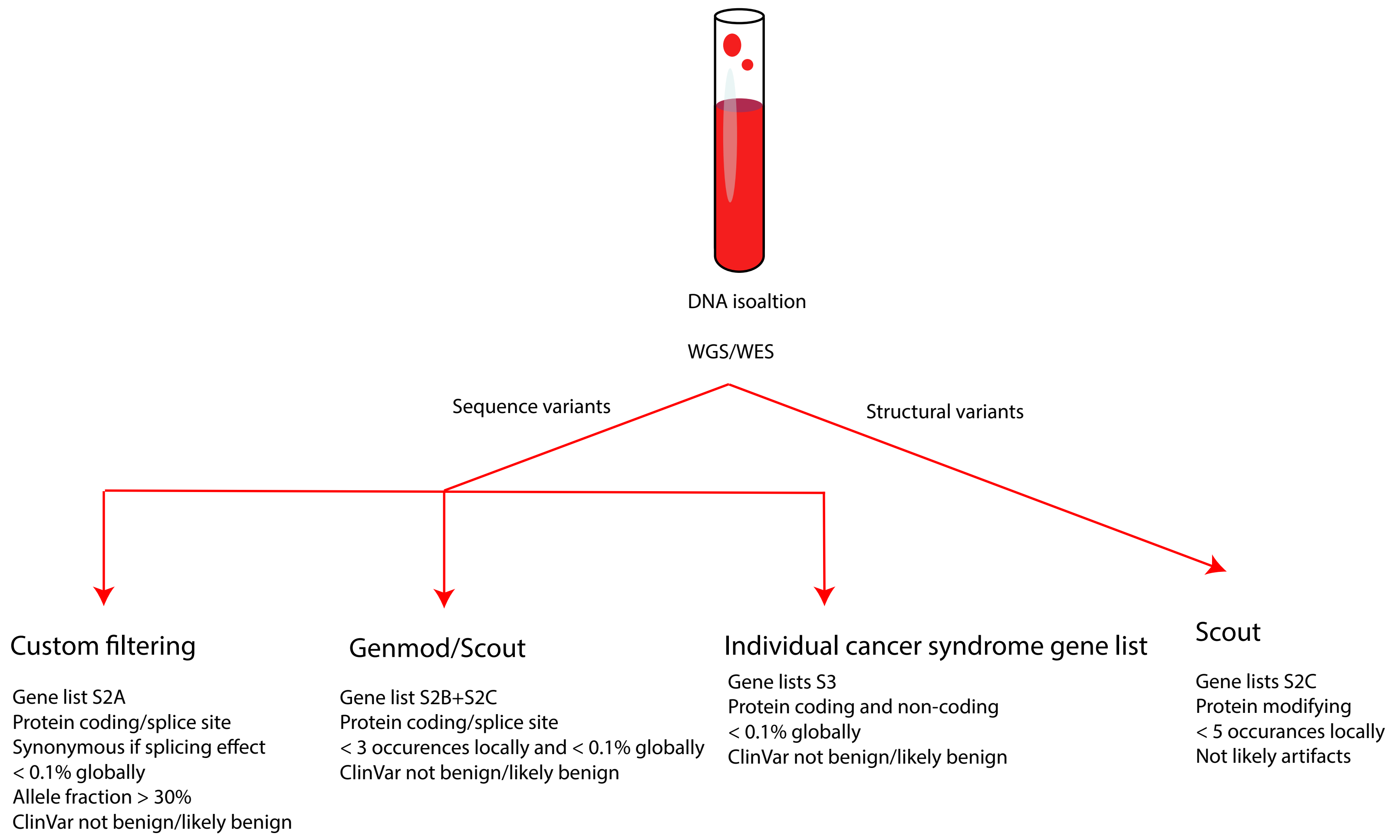
DNA isoaltion
WGS/WES
Sequence variants
Structural variants
Custom filtering
Gene list S2A
Protein coding/splice site
Synonymous if splicing effect
< 0.1% globally
Allele fraction > 30%
ClinVar not benign/likely benign
Genmod/Scout
Gene list S2B+S2C
Protein coding/splice site
< 3 occurences locally and < 0.1% globally
ClinVar not benign/likely benign
Individual cancer syndrome gene list
Gene lists S3
Protein coding and non-coding
< 0.1% globally
ClinVar not benign/likely benign
Scout
Gene lists S2C
Protein modifying
< 5 occurances locally
Not likely artifacts
